# Development of Ecofriendly Derivative Spectrophotometric Methods for the Simultaneous Quantitative Analysis of Remogliflozin and Vildagliptin from Formulation

**DOI:** 10.3390/molecules26206160

**Published:** 2021-10-12

**Authors:** Mahesh Attimarad, Katharigatta N. Venugopala, Bandar E. Al-Dhubiab, Rafea Elamin Elgack Elgorashe, Sheeba Shafi

**Affiliations:** 1 Department of Pharmaceutical Sciences, College of Clinical Pharmacy, King Faisal University, Al Hofuf 31982, Saudi Arabia; kvenugopala@kfu.edu.sa (K.N.V.); baldhubiab@kfu.edu.sa (B.E.A.-D.); 2 Department of Biotechnology and Food Science, Faculty of Applied Sciences, Durban University of Technology, Durban 4000, South Africa; 3 Department of Chemistry, College of Science, King Faisal University, Al Hofuf 31982, Saudi Arabia; relgorashe@kfu.edu.sa; 4 Department of Nursing, College of Applied Medical Sciences, King Faisal University, Al Ahsa 31982, Saudi Arabia; sheeba@kfu.edu.sa

**Keywords:** vildagliptin, remogliflozin, ratio derivative spectrophotometry, determination, formulation, ecofriendly

## Abstract

Three rapid, accurate, and ecofriendly processed spectrophotometric methods were validated for the concurrent quantification of remogliflozin (RGE) and vildagliptin (VGN) from formulations using water as dilution solvent. The three methods developed were based on the calculation of the peak height of the first derivative absorption spectra at zero-crossing points, the peak amplitude difference at selected wavelengths of the peak and valley of the ratio spectra, and the peak height of the ratio first derivative spectra. All three methods were validated adapting the ICH regulations. Both the analytes showed a worthy linearity in the concentration of 1 to 60 µg/mL and 2 to 90 µg/mL for VGN and RGE, respectively, with an exceptional regression coefficient (r2 ≥ 0.999). The developed methods demonstrated an excellent recovery (98.00% to 102%), a lower percent relative standard deviation, and a relative error (less than ±2%), confirming the specificity, precision, and accuracy of the proposed methods. In addition, validated spectrophotometric methods were commendably employed for the simultaneous determination of VGN and RGE from solutions prepared in the laboratory and the formulation. Hence, these methods can be utilized for the routine quality control study of the pharmaceutical preparations of VGN and RGE in pharmaceutical industries and laboratories. The ecofriendly nature of the anticipated spectrophotometric procedures was confirmed by the evaluation of the greenness profile by a semi-quantitative method and the quantitative and qualitative green analytical procedure index (GAPI) method.

## 1. Introduction

**Figure 1:**
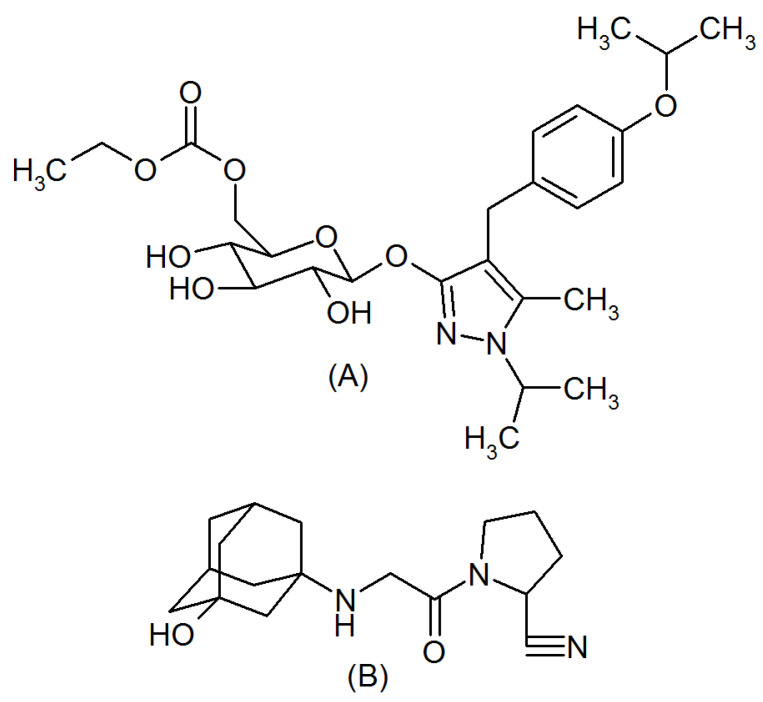
Chemical structure of remogliflozin etabonate (**A**) and vildagliptin (**B**).

Diabetes mellitus (DM) is a metabolic disease characterized by elevated blood glucose levels. It is estimated that one out of eleven people suffer from DM globally, and 90% of the population has type 2 DM (T2DM). Experts predict that the occurrence of cases of DM will increase to nearly 642 million by the end of 2040 from the current levels (422 million at present). The occurrence of T2DM is becoming common in the elderly population due to stress and physical inactivity. However, obesity, food habits, and hereditary factors predispose diabetes among the younger population [1,2]. The pathophysiological mechanisms and treatment of diabetes are complicated; hence, a multiple intervention approach such as the practice of healthy diets, physical activity, and various therapeutic strategies may help to minimize the complications of diabetes. Recently developed dipeptide peptidase-4 (DPP-4) and sodium-glucose transporter-2 (SGLT-2) inhibitors showed an enhanced HbA1c control when compared with conventional sulfonylureas and thiazolidinediones [3]. The Food and Drug Administration (FDA) has approved a fixed-dose remogliflozin and vildagliptin tablet for T2DM. Remogliflozin etabonate (Figure 1A, RGE) is an oral hypoglycemic drug [4], which acts by inhibiting the SGLT-2 enzyme and thereby decreasing the reabsorption of glucose from the glomerular filtrate back to the blood. SGLT-2 inhibitors reduce cardiovascular events, body weight, and also show a defensive effect on the renal system. These functional properties of SGLT-2 inhibitors considerably reduce the hospitalization of T2DM patients exclusively due to heart failure [5,6]. Vildagliptin (Figure 1B, VGN), a DPP-4 inhibitor, decreases the blood sugar level by protecting the incretins from degradation, which helps in the production of insulin after food and reduces glucagon formation in the liver. The protection of incretins also helps in reducing body weight by decreasing the appetite and prolonging the slow digestion of food [7,8].

Few quantitative analytical procedures are illustrated in the literature for the analysis of RGE and VGN alone and with metformin from medicines and biological fluids. A quantitative determination of RGE alone was explained using UV-Vis spectrophotometry [9,10], HPLC [11,12], and LCMS [13].Derivative UV spectrophotometry, RP-HPLC, and UPLC procedures were stated for the concurrent estimation of RGE with metformin [14,15,16].

Different analytical methods have been reported in the literature for the quantification of VGN from formulations and biological samples. VGN alone was determined using UV-Vis spectrophotometry [17,18,19], HPTLC [20], and UPLCMS [21]. Several spectrophotometric [22,23,24] and HPLC [24,25,26,27] methods were reported for the determination of VGN along with other drugs.

Several analytical methods have been reported for the determination of VGN and RGE alone and with other active ingredients. However, no quantitative analytical method has been described for the concurrent estimation of VGN and RGE from a formulation. Derivative UV spectrophotometric techniques are simple, accurate, fast, and may possibly be utilized for the quantification of multicomponent formulations showing overlapping spectra [28,29,30,31,32]. Hence, in the current work, three spectrophotometric methods were validated and applied to a concurrent determination of VGN and RGE from laboratory mixed solutions and formulations. Water was used as a dilution solvent for the samples, making the developed spectrophotometric methods ecofriendly.

## 2. Results and Discussion

**Figure 2:**
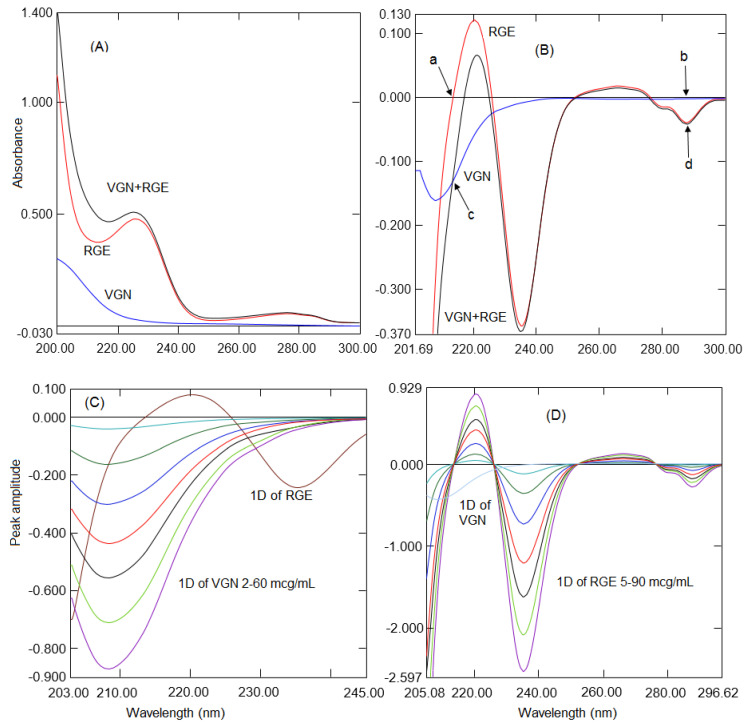
(**A**) Normal spectra of RGE, VGN, and a mixture. (**B**) First derivative spectra of RGE, VGN, and the mixture. Zero-crossings of RGE (a), zero-crossings of VGN (c), and the same amplitude points (b and d). (**C**) First derivative spectra of VGN 2 to 60 µg/mL with the RGE spectrum. (**D**) First derivative spectra of RGE 5 to 90 µg/mL with the VGN spectrum.

UV-Vis spectroscopic methods are extensively used analytical techniques due to their simplicity, accuracy, and reproducibility. Salinas et al. reported a derivative spectroscopic method [29] for the analysis of multicomponent formulations with overlapping spectra (Figure 2A), which cannot be analyzed by a direct UV measurement. Many reports have demonstrated the use of derivative spectroscopic methods for the analysis of multicomponent formulations without a prior separation [28,29,30,31,32]. RGE showed UV absorption in the range of 200 to 300 nm due to the presence of individual five and six membered rings whereas VGN showed below 230 nm due to an absence of an aromatic ring and the presence of an aliphatic nitrile and carbonyl group. However, both analytes did not show any absorption above 300 nm; hence, in the present work, the analytes were scanned in the wavelength rage of 200 to 300 nm. In the present work, three processed UV spectroscopic procedures were validated for the concurrent quantification of VGN and RGE. The first procedure was established on the measurement of absorption at zero-crossings of one of the analytes where another analyte had a degree of absorption. For the determination of VGN and RGE, normal absorption spectra were processed into first derivative spectra utilizing 4 nm as ∆λ and a scaling factor of 10. Different wavelengths of 2, 4, 8, and 10 nm were envisaged during the first derivative spectrum; however, 4 nm resulted in smooth spectra so 4 nm was selected. A scaling factor of 10 demonstrated a sufficient peak amplitude at a low concentration of VGN; hence, a scaling factor of 10 was selected. The first derivative spectra of VGN showed a good absorption at 213.7 and 225 nm where RGE had zero absorption (Figure 2B). Similarly, RGE showed good absorptions at 241.5, 265.7, and 287.7 nm where VGN had no absorption. However, at 213.7 and 287.7 nm, VGN and RGE showed a good recovery and reproducibility, respectively. The comparison of the first derivative spectra obtained from the pure and the combination of VGN and RGE showed the same amplitude. Hence, the calibration curves for the determination of VGN and RGE were computed by evaluating the peak height at 213.7 nm (Figure 2C) and 287.7 nm, respectively (Figure 2D).

### 2.1. Ratio Difference Absorption Method

**Figure 3:**
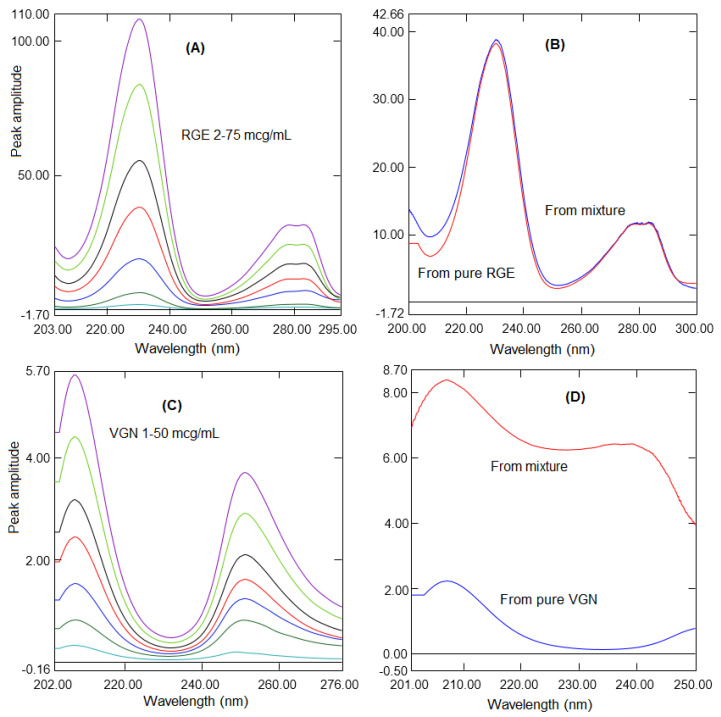
(**A**) Ratio absorption spectra of RGE (2 to 75 µg/mL) using a 5 µg/mL spectrum of VGN. (**B**) Ratio absorption spectra of pure RGE and a mixture. (**C**) Ratio absorption spectra of VGN (1 to 50 µg/mL) using a 5 µg/mL spectrum of RGE. (**D**) Ratio absorption spectra of pure VGN and a mixture.

The solutions of RGE and VGN were subjected to UV absorption in the wavelength range of 200 to 300 nm. The selection of the divisor analyte concentration is important in developing ratio spectra; hence, different concentrations of spectra were tried. However, no significant difference was observed in terms of the linearity range with different concentrations although a good intensity of ratio spectra and reproducibility were observed with 5 µg/mL spectra as the divisor. Hence, 5 µg/mL spectra of RGE and VGN were envisaged as the divisor spectra. For the estimation of REM, a mixture spectra consisting of 2 to 75 µg/mL of REM were divided with a 5 µg/mL spectrum of VGN to generate the ratio spectra for REM (Figure 3A). The peak amplitude differences were calculated by subtracting the peak amplitude at 230.3 and 251.4 nm, and linearity curves were generated against the corresponding concentration. Figure 3B shows the same peak height from the ratio spectra generated from the combination (REM and VGN). The pure REM indicated that REM could be determined in the presence of another analyte, VGN. Similarly, a mixture of REM and VGN consisting of 2 to 50 µg/mL of VGN was divided with a 5 µg/mL spectrum of RGE to generate the ratio spectra for VGN (Figure 3C). The two wavelengths selected were 207.2 and 230.6 nm, the peak amplitude difference was calculated, and the linearity curve was generated by drawing a graph against the respective concentration. Figure 3D shows the same peak amplitude in the ratio spectra generated from the mixture and the pure VGN spectra, demonstrating the application of the ratio difference absorption method for the quantification of VGN in the presence of RGE.

### 2.2. Ratio First Derivative Method

**Figure 4:**
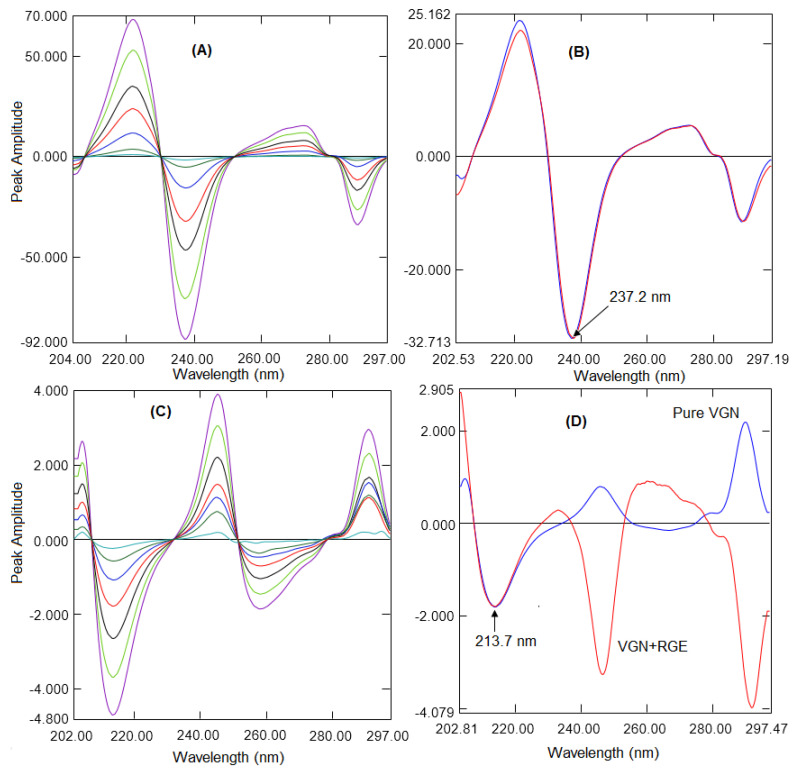
(**A**) Ratio first derivative spectra of RGE (2 to 75 µg/mL). (**B**) Ratio first derivative spectra of pure RGE and a mixture. (**C**) Ratio first derivative spectra of VGN (1 to 50 µg/mL). (**D**) Ratio first derivative spectra of pure VGN and a mixture.

From the equation P_A_ = P_R_ + δ (Materials and Methods), the constant (δ) could be excluded by changing the ratio spectrum into the derivative spectrum. The conversion of the derivative spectrum generated serval maxima and minima. The peak amplitude was proportional to the amount and the effects of another analyte and tablet excipients were excluded. This was a substitute technique to the ratio difference method to quantify VGN in the presence of RGE and vice versa. For the quantification of VGN, the ratio spectra of VGN were changed into first derivative spectra by applying 4 nm as ∆λ and a scaling factor of 10 (Figure 4A). Various wavelengths between 2 and 8 nm were tried as ∆λ although 4 nm exhibited an enhanced sensitivity. Three maxima at 204.4, 245.4, and 289.7 nm and two minima peaks at 213.7 and 257.7 nm were observed in the first derivative spectra of VGN. The peak amplitude was good at these wavelengths; however, the peak height was better at 213.7 nm. Hence, 213.7 nm was selected to generate the calibration curve. The same peak amplitudes were observed at 213.7 nm in the first derivative spectra generated from the mixture and the pure VGN spectra (Figure 4B). Similarly, for the quantification of RGE, the ratio absorption spectra of RGE were processed to develop first derivative spectra with 4 nm as ∆λ and a scaling factor of 10 (Figure 4C). Two maxima at 221.8 and 272.6 nm and two minima peaks at 237.2 and 288.2 nm were observed, respectively, in the first derivative spectra of RGE. The reproducibility and sensitivity were better at 237.2 nm; hence, 237.2 nm was selected to construct the calibration curve. The same peak amplitudes were observed at 237.2 nm in the first derivative spectra generated from the mixture and the pure RGE spectra (Figure 4D).

### 2.3. Method Validation

Optimized spectroscopic methods were validated in terms of linearity, the limit of detection/quantification, accuracy, interday and intraday precision, selectivity, and stability of the standard solutions under experimental and storage conditions.

#### 2.3.1. Linearity Range

The calibration curve was constructed using seven solutions of analytes using a series of solutions comprising 2 to 60 µg/mL for VGN and 5 to 90 µg/mL for RGE in triplicate by the first derivative spectroscopic method. The linearity was established in the range of 1 to 50 µg/mL for VGN and 2 to 75 µg/mL for RGE by the ratio difference and ratio first derivative methods. Both analytes did not show any absorption above 300 nm; hence, the solutions were scanned between the wavelengths of 200 to 300 nm. The molar absorptivity of the normal spectra of VGN and RGE was found to be 18,100.24 L M^−1^ cm^−1^ at 209.2 nm and 32,133.33 L M^−1^ cm^−1^ at 225.9 nm, respectively. The peak amplitudes were measured from the first derivative, ratio derivatives, and first derivative of the ratio spectra and calibration curves were constructed between the peak amplitudes and the corresponding concentration (Supplementary File S1). The linearity equations and coefficients are presented in Table 1. The intercept values for the RGE curves were negative, indicating that the model overestimated the average absorption; hence, there was a requirement to subtract the predicted concentrations. The intercept values for the VGE curves were positive, indicating that the model underestimated the average absorption; hence, there was a requirement for the predicted concentrations to be added.

#### 2.3.2. Accuracy

The accuracy of the optimized methods was performed by evaluating VGN and RGE at three different concentrations (low, medium, and high) covering the complete linearity concentration. The concentration of analytes was computed from the linearity equations generated from the three methods. The accuracy of the methods was presented as a percentage recovery and a percent relative error and is presented in Table 1. The mean percent recovery was found to be 99.13% to 100.82% for RGE and 99.52% to 101.07% for VGN, indicating the accuracy of the developed spectroscopic methods. The percent recovery was between 98.00% and 102.00%, indicating the accuracy of the anticipated procedures.

#### 2.3.3. Precision

The reproducibility of the optimized procedures was assessed by performing an intraday analysis using three different concentrations comprising the complete linearity concentration. The solutions were analyzed on the same day in triplicate and the percent relative standard deviation (%RSD) was calculated. The intermediate precision of the procedures was performed by evaluating the above-prepared solution on three successive days. The interday precision was expressed as a percent relative standard deviation and is tabulated in Table 1. The intraday precision for VGN ranged from 0.610 to 0.871 and it was from 0.730 to 1.843 for RGE whereas the interday precision ranged from 0.725 to 1.748 for VGN and 0.611 to 1.383 for RGE. The %RSD was lower than 2 in all cases, representing the good precision of the UV derivative spectroscopic methods.

#### 2.3.4. Limit of Detection and Quantification

One of the LOD and LOQ methods utilized the standard deviation of the intercept and slope of the calibration curve, which was 3.3 times the SD of the intercept/slope of the linearity curve for the LOD and 10 times the SD of the intercept/slope of the calibration curve for the LOQ. The LOD and LOQ determined for all three methods are tabulated in Table 1. Low LOQ values indicated the sensitivity of the anticipated procedures.

#### 2.3.5. Specificity

Different ratios of VGN and RGE within the linearity range were prepared in the laboratory and investigated for testing the specificity of the proposed methods. The obtained outcomes by all three methods are tabulated in Table 2. The good analysis outcomes with a lower percent relative standard deviation confirmed the specificity of the optimized spectroscopic methods.

### 2.4. Application of the Optimized Methods for the Formulation

The proposed UV derivative spectroscopic methods were utilized for the simultaneous quantification of VGN and RGE in the formulation. The results, as presented in Table 2, confirmed the accuracy of the quantification of the analytes with a good agreement with the labeled quantity of the active ingredients of the medicine and confirmed the nonexistence of the influence of the formulation excipients on the assay of both analytes. The excipients used for preparation of the tablets were common and, in general, present in the formulation. The proposed methods eliminated the effects of the excipients; hence, they could be used for quality control.

The accuracy of the optimized procedures was further confirmed using the standard addition method. An assessed quantity of VGN and RGE was transformed to the previously evaluated formulation solution and the spiked solution was analyzed by all three proposed methods. The achieved results are tabulated in Table 3. The mean percent recovery (100 ± 2%) and the relative standard deviation (<2%) were well within the acceptable range, assuring the adequate accuracy of the proposed procedures.

### 2.5. Greenness Evaluation of the Optimized Methods

**Figure 5:**
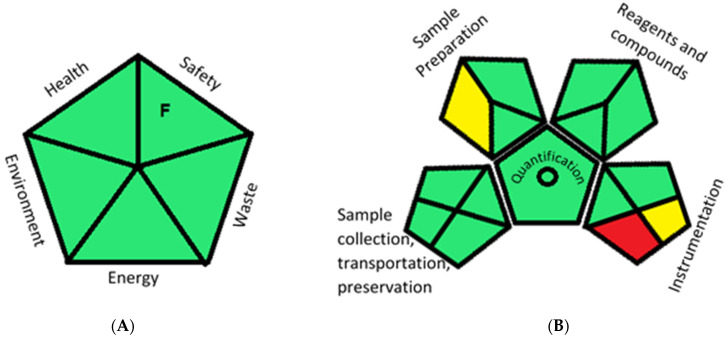
(**A**) Greenness evaluation results of the proposed method by Raynie et al.’s method [33]. (**B**) The GAPI method.

The development of ecofriendly analytical methods is a requirement of the present day to save the environment. In the present work, two greenness evaluation methods were implemented to evaluate the ecofriendly nature of the proposed methods. Raynie et al. [33] developed a semi-quantitative method for a greenness evaluation (Supplementary material Table S1) that considered the nature of the chemicals and instruments used in the experiment as these are the main contributors to environmental pollution. In the current work, ethanol was used as a solvent to dissolve one of the analytes and water was used for further dilation, generating less than 5% alcohol in each solution. In addition, due to the use of dilute ethanol, the evaporation of ethanol was negligible. The measurement was made using a spectrophotometer and a computer; therefore, the energy consumed during the process was safe. Each solution prepared for the analysis contained less than 5% ethanol and a few micrograms of the drugs, generating less than 50 g of waste. Hence, the greenness profile (Figure 5A) of all three proposed spectroscopic methods was ecofriendly. The second method adopted for the greenness evaluation was based on the quantitative and qualitative method developed by Plotka-Wasylka, known as the green analytical procedure index (GAPI) [34]. The GAPI involves the grading of 15 parameters of analytical methods, starting from the sample preparation and the hazardous nature of the chemicals, solvents and instruments used along with the nature of the analysis to the waste treatment of the samples after the completion of experiments [35] (Supplementary material Table S2). The proposed spectroscopic methods involved a simple sample preparation during the analysis; hence, there was no need for the storage and transportation of the sample. A direct sample preparation technique was used during the sample preparation using green solvents without any extraction and pretreatment processes. Apart from water, another solvent used in the optimized methods was ethanol, which is considered to be a safe solvent with an NFPA irritant and the flammable score of one with no hazardous effect on animals and the environment. The total volume of each sample was 5 mL. A waste treatment procedure had not been established; hence, the parameter color codes for 14 and 15 were marked as yellow and red. However, the overall profile of the GAPI (shown in Table 4 and Figure 5B) confirmed the ecofriendly nature of the optimized spectroscopic methods.

## 3. Materials and Methods

### 3.1. Instruments

The spectrophotometric determination was carried out using a UV-Vis spectrophotometer 1600 (Shimadzu, Japan) using 1 cm quartz cuvettes. The samples were scanned in the fast mode with a slit width of 0.1 nm. The processing of the scanned spectra was completed with UV-probe Ver 2.21 software (Shimadzu, Japan).

### 3.2. Chemicals

Standard drugs of VGN and RGE were purchased from Biokemics (Hyderabad, India). A fixed-dose combination of VGN (50 mg) and RGE (100 mg) was not available in the local pharmacies; hence, a geometrically mixed tablet was prepared by mixing a sufficient quantity of VGN and RGE with solid dosage form excipients of microcrystalline cellulose, hydroxypropyl cellulose, crospovidone, colloidal silicon dioxide, and stearic acid. Analytical grade ethanol was bought from Sigma Aldrich (St. Louis, MO, USA). Purified water prepared using a double-distil water purifier in our laboratory was utilized throughout the analysis.

### 3.3. Preparation of the Standard Solutions

The stock solutions of VGN and RGE were prepared by directly transferring 100 mg of VGN and RGE in 100 mL graduated flasks separately. VGN was dissolved using water and RGE was dissolved using ethanol to obtain a 1000 µg/mL solution. For preparing the calibration standards and the solutions for validation, the stock solutions were diluted with water by maintaining 200 µL of ethanol per sample in a total volume of 5 mL. A blank solution was prepared by adding 200 µL of ethanol in 5 mL of water.

### 3.4. Preparation of the Sample Solutions

The newly approved solid dosage forms of VGN (50 mg) and RGE (100 mg) were not accessible in the local pharmacies. Hence, a table mixture was prepared in the laboratory by mixing a sufficient quantity of VGN and RGE with tablet excipients (microcrystalline cellulose, hydroxypropyl cellulose, crospovidone, colloidal silicon dioxide, and stearic acid). A thoroughly mixed tablet powder consisting of 50 mg of VGN and 100 mg of RGE was transferred into a 100 mL graduated flask. A total of 25 mL of ethanol was transferred and sonicated for 15 min to completely extract both active ingredients. The solution was filtered into another flask, the residue was washed with fresh ethanol, and the final volume was attuned by adding a sufficient amount of ethanol. For the analysis of the solutions, the concentration of the sample solutions was brought in the range of the calibration curve using distilled water.

### 3.5. Theory of the Ratio Derivatization Technique

This was a simple and specific method adopted by Lotfy and Hegazy [36] for the simultaneous determination of a binary mixture showing complete overlap spectra without any separation. It was established by generating the ratio spectra by dividing the mixture spectra (VR) with one of the analyte spectra (R’ or V’) and determining the peak amplitude difference at two designated wavelengths, which was directly related to the concentration. According to the Beers law absorption of the mixture of components, A_VR_ is represented by Equation (1).

A_VR_ = Ɛ_V_ × C_V_ + Ɛ_R_ × C_R_ (1)

where Ɛ_V_ and Ɛ_R_ are the molar extinction coefficients of VGN and RGE, respectively, at a particular wavelength whereas C_V_ and C_R_ are concentrations of VGN and RGE, respectively. The ratio spectra were generated by dividing the mixture spectra (VR) with an analyte spectrum (Av’ = Ɛ_V’_× C_V’_) at the concentration V’ to determine another analyte (R), which is represented by Equation (2):

A_VR/_A_v’_ = C_V_/C_V’_ + A_R_/A_V’_. (2)

The C_V_/C_V’_ is constant (δ); hence, the above equation can be simplified to:

P_A_ = P_R_ + δ (3)

where P_A_ is the A_VR/_A_v’_ absorption of the ratio spectra of a mixture of components to one of the components whereas P_R_ is the absorbance of the ratio spectra of one component to another component.

From Equation (3), the constant δ can be eliminated by determining the change in absorbance at two selected wavelengths from the ratio spectra as per Equation (4):

P_A1_ − P_A2_ = (P_R1_ + δ) − (P_R2_ + δ). (4)

Equation (4) can then be simplified to Equation (5):

ΔP = P_R1_ + P_R2_ (5)

where P_R1_ and P_R2_ are peak amplitudes at wavelengths λ_1_ and λ_2_ of the ratio spectra. Equation (5) represents the absorption of only one analyte (RGE), eliminating the effects of the additional analyte (VGN). In the current effort, the mixture of RGE and VGN was prepared in the calibration curve range and the spectra were recorded.

### 3.6. Procedure

#### 3.6.1. First Derivative Spectrophotometric Method (FDS)

A sufficient quantity of stock solutions of VGN and RGE was measured into 5 mL volumetric flasks separately to obtain the concentrations of 2, 10, 20, 30, 40, 50, and 60 µg/mL of VGN and 5, 15, 30, 45, 60, 75, and 90 µg/mL of RGE. The solutions were exposed to UV absorption using ethanol–water as a blank solution and the spectra were deposited into the computer. The RGE absorption spectra were transformed into first derivative spectra with 4 nm as ∆λ and a scaling factor of 10. The peak amplitude was measured at 287.7 nm and the linearity curve was created against the respective concentration. VGN spectra were also transformed into first derivative spectra with 4 nm as ∆λ and a scaling factor of 10. The peak height was measured at 213.7 nm and the linearity curve was developed using the respective concentrations. In addition, regression equations and regression coefficients were constructed from both curves.

#### 3.6.2. Ratio Absorption Difference Method (RAD)

Standard solutions of VGN and RGE consisting of 5 µg/mL were prepared separately. A sufficient amount of standard solutions of VGN and RGE was transferred into seven 5 mL graduating flasks to obtain concentrations in the range of 1–50 µg/mL and 2–75 µg/mL. All the solutions were subjected to UV absorption at 200–300 nm and stored in the computer. The scanned spectra of the mixture were subjected to a division by the spectrum of RGE to generate the ratio spectra of VGN in the range of 1–50 µg/mL, which were smoothened using 4 nm and stored in the computer. The peak amplitude discrepancy was calculated by subtracting the peak amplitudes at 207.2 nm from 230.6 nm. Similarly, the mixture spectra were subjected to a division by the spectrum of VGN to generate the ratio spectra of RGE with concentrations of 2–75 µg/mL. The peak amplitude difference was recorded for each spectrum by evaluating the peak amplitudes at 230.3 and 251.4 nm. The calibration curves were constructed for both analytes by drawing a graph between the difference in the peak height and the respective concentration. The regression equations and coefficients were computed from the linearity curves.

#### 3.6.3. Ratio First Derivative Absorption Method (RFA)

The above-recorded ratio spectra of VGN were modified into first derivative spectra utilizing 4 nm as ∆λ and a scaling factor of 10. The peak height was calculated at 213.7 nm and the linearity curve was created by drawing a graph between the peak height and the corresponding concentration. Similarly, the ratio spectra of RGE were processed into first derivative spectra utilizing 4 nm as ∆λ and a scaling factor of 10. The peak height of the spectra was measured at 237.2 nm and the linearity curve was created against the corresponding concentration.

#### 3.6.4. Application of the Optimized Methods to the Formulation

The above-prepared formulation solution was diluted to obtain the amount of analytes in the range of the linearity range. The solutions were subjected to UV absorption at 200–300 nm and stored in the computer. For the FDS method, the stored spectra were transferred into first derivative spectra utilizing 4 nm as ∆λ and a scaling factor of 10. The concentration of RGE was computed by measuring the peak height of the first derivative spectra at 287.7 nm and using a regression equation. The peak height was calculated at 213.7 nm for VGN and the amount of analyte was estimated by means of a regression equation. For the ratio difference, the absorption method scanned spectrum of the formulation was divided by the VGN spectra to generate the RGE ratio spectra; similarly, the formulation spectrum was divided by the RGE spectrum to generate the VGN ratio spectra followed by smoothening with a wavelength of 4 nm. The peak amplitude variance was calculated by deducting the peak height at 207.2 nm from 230.6 nm for computing the concentration of VGN using the corresponding regression equation. Similarly, the peak amplitude difference was computed at 230.3 nm and 251.4 nm for determining the concertation of RGE. The above-generated ratio spectra of VGN and RGE were processed into first derivative spectra utilizing 4 nm as ∆λ and a scaling factor of 10. The peak height was measured at 213.7 nm for VGN and at 237.2 nm for RGE. The amounts of VGN and RGE were determined from the respective linearity equations.

## 4. Conclusions

Despite the complete overlapping of both analytes, derivative spectrophotometric methods were successfully developed for the concurrent estimation of VGN and RGE from the combined medicine. The proposed methods were simple, accurate, rapid, and validated according to the ICH guidelines. The greenness profile, evaluated by two different methods, confirmed the ecofriendly nature of the optimized spectroscopic methods. All three methods were utilized for a simultaneous determination without interference from the formulation excipients; hence, these procedures could be useful for the consistent quality control of medical dosage forms comprising VGN and RGE. In addition, the reported methods are considered to be economical as there was no need of expensive solvents and instruments in chromatographic methods. No additional software was purchased because the software provided with the UV spectrophotometer was used.

## Figures and Tables

**Table 1:** Validation parameter results of the proposed spectroscopic methods for the simultaneous determination of VGN and RGE.

Validation Parameters	Remogliflozin			Vildagliptin		
	FDS	RDS	RFD	FDS	RDS	RFD
Wavelength (nm)	287.7	230.3–251.4	237.2	213.7	207.2–230.6	213.7
Linearity range (µg/mL)	5–90	2–75	2–75	2–60	1–50	1–50
Slope	0.0031	1.328	1.226	0.012	0.0983	0.0920
Intercept	−0.0051	−1.221	−0.884	0.008	0.2489	0.0841
Regression coefficient (r^2^)	0.9997	0.9998	0.9997	0.9996	0.9990	0.9994
LOD (µg/mL)	1.361	0.583	0.408	0.484	0.272	0.176
LOQ (µg/mL)	4.126	1.768	1.236	1.469	0.827	0.535
Accuracy (Mean % ± SD)	99.41 ± 1.170	99.13 ± 0.665	100.82 ± 0.910	99.52 ± 0.834	99.88 ± 1.655	101.07 ± 0.729
Precision (%RSD)						
Intraday	0.730	1.026	1.843	0.610	0.871	0.728
Interday	0.937	1.383	0.611	1.748	0.725	1.287

**Table 2:** Assay results of the laboratory mixed solutions of VGN and RGE.

Laboratory Prepared Mixture (µg/mL)		Remogliflozin (% Recovery)			Vildagliptin (% Recovery)		
RGE	VGN	FDS	RDS	RFD	FDS	RDS	RFD
10	50.00	99.20	101.60	98.50	98.30	98.22	101.04
40	50.00	97.93	100.70	99.43	98.58	99.06	100.78
40	5.00	100.30	96.48	100.20	98.60	99.40	99.80
70	5.00	99.24	98.06	99.03	101.20	100.40	98.60
70	25.00	100.67	98.73	98.21	99.24	99.04	101.36
Across Mean		99.47	99.11	99.07	99.18	99.27	100.32
SD		0.964	1.840	0.702	1.054	0.705	1.004

**Table 3:** Assay results of the formulation and the standard addition method results.

Formulation Concentration		Remogliflozin (Mean %± SD)			Vildagliptin (Mean % ± SD)		
RGE	VGN	FDS	RDS	RFD	FDS	RDS	RFD
100 mg	50 mg	99.42 ± 0.985	99.62 ± 0.788	99.10 ± 0.76	99.77 ± 1.563	100.04 ± 1.384	100.19 ± 0.797
Standard Addition Method							
Amount Added (µg/mL)		Remogliflozin (% Recovery)			Vildagliptin (% Recovery)		
10 µg/mL	5	98.70	100.70	98.30	99.20	100.40	99.80
20 µg/mL	10	100.95	98.85	99.05	101.90	98.20	101.30
30 µg/mL	15	96.47	99.30	100.23	98.20	101.53	99.47
Across Mean		98.71	99.62	99.19	99.77	100.04	100.19
%RSD		1.830	0.788	0.796	1.563	1.384	0.797

**Table 4:** The GAPI evaluation results for the spectrophotometric methods.

Category	UV Spectrophotometric Methods
Sample Preparation	
Collection (1)	In-line
Preservation (2)	Nil
Transport (3)	Nil
Storage (4)	Nil
Type of method: direct or indirect (5)	Direct (no sample preparation)
Scale of extraction (6)	Nil
Solvents/reagents used (7)	Green Solvents
Additional treatments (8)	Nil
Reagent and solvents	
Amount (9)	<10 mL
Health hazard (10)	Ethanol, slightly toxic and irritant NFPA score 1
Safety hazard (11)	Ethanol, instability score 0, flammability score 1, no special hazard
Instrumentation	
Energy (12)	≤0.1 kWh/sample
Occupational hazard (13)	Hermetic sealing of the analytical procedure
Waste (14)	1–10 mL
Waste treatment (15)	No treatment
Quantification	Yes

## Data Availability

The data generated during this work were included in the manuscript and submitted as Supplementary files.

## Supplementary Materials

The following are available online. Figure S1: UV derivative spectra of VGN and RGE of laboratory mixed solutions. Figure S2: UV derivative spectra of VGN and RGE for the formulation and standard addition method. File S1: Calibration curves of analytes by UV derivative methods. Table S1: Green assessment profile proposed by Raynie et al. Table S2: Green analytical procedure index parameters.
