# Supplementary material for: Development of Ecofriendly Derivative Spectrophotometric Methods for the Simultaneous Quantitative Analysis of Remogliflozin and Vildagliptin from Formulation
Source: Molecules. 2021 Oct 12;26(20):6160. doi: 10.3390/molecules26206160 (PMC8537597; doi:10.3390/molecules26206160)
Supplement: Supplementary file 1 [file molecules-26-06160-s001.zip › molecules-1397718-supplementary/Suplementary file/Figure S1.pdf]

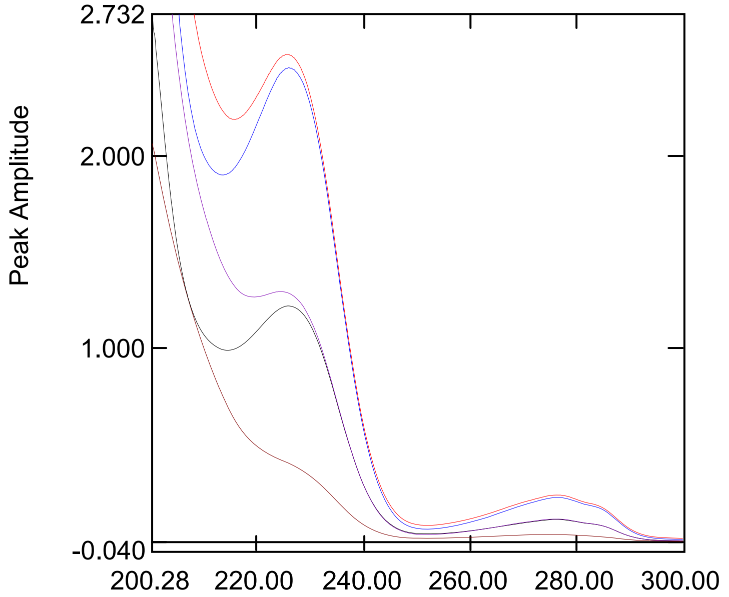

UV absorption spectra of Lab mixed solution

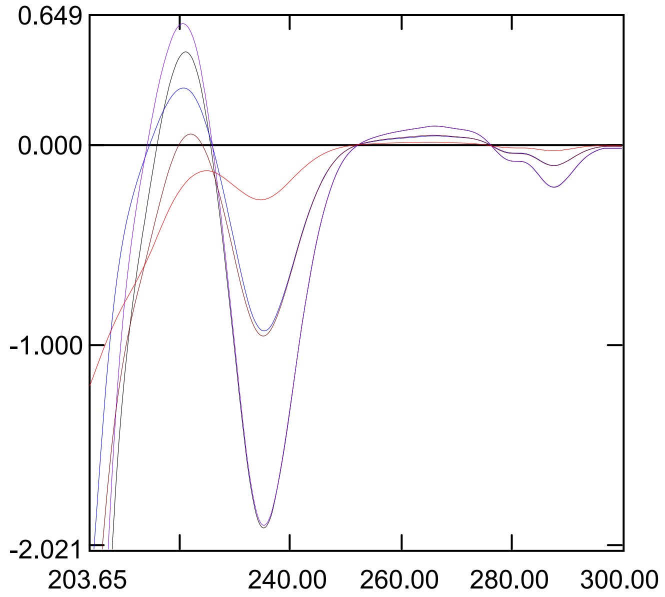

First derivative UV absorption spectra of Lab mixed solution

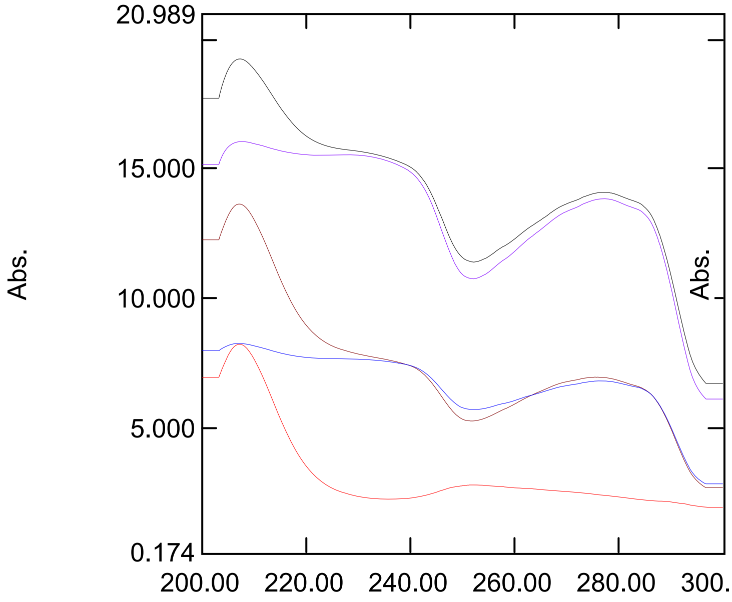

Ratio absorpeion sepctra of VGN Lab mixed solution using as divisor 5mcg/ml of REM

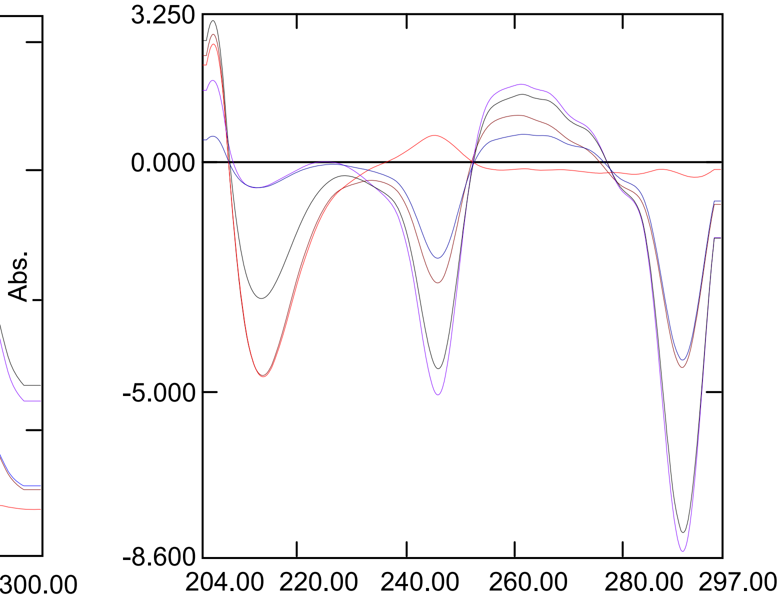

Ratio first derivative sepctra of VGN Lab mixed solution using 5mcg/ml of REM as divisor

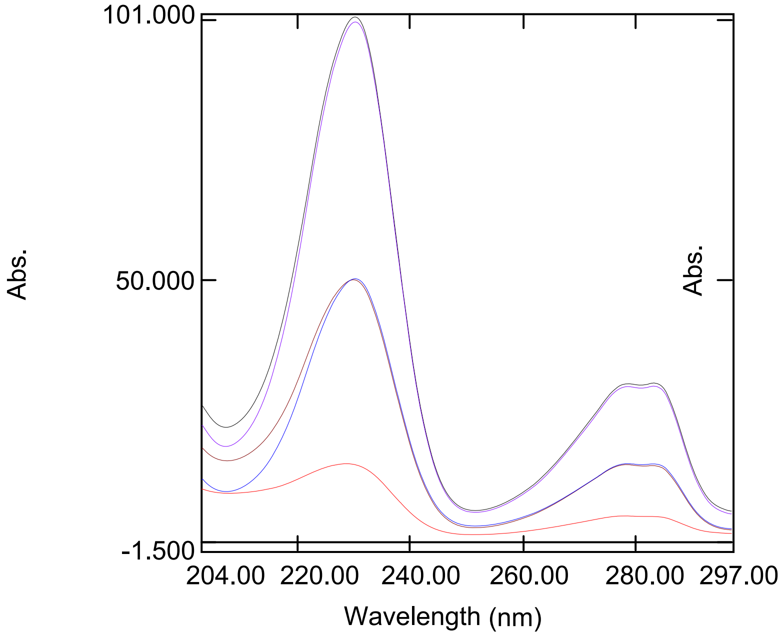

Ratio absorpeion sepctra of RGE Lab mixed solution using 5mcg/ml of VGN as divisor

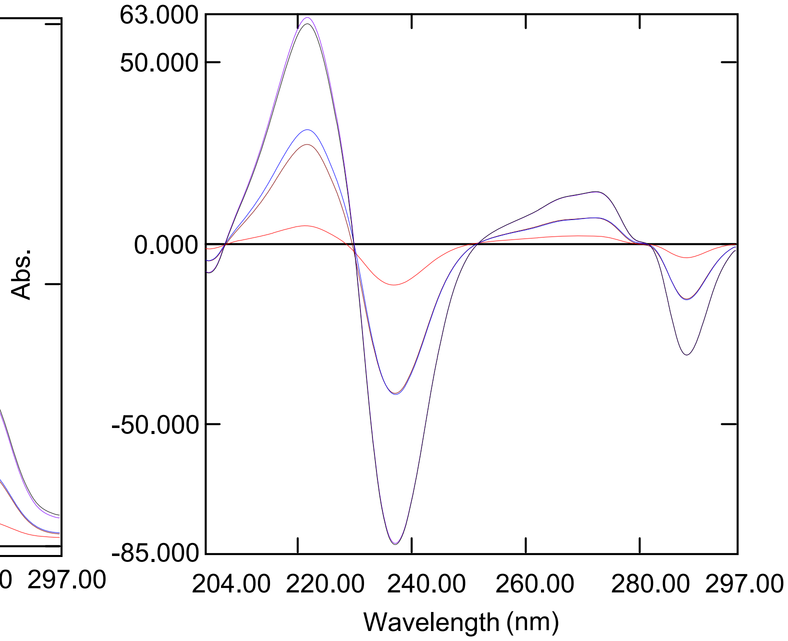

Ratio first derivative sepctra of RGE Lab mixed solution using 5mcg/ml of VGN as divisor
