# Supplementary material for: Development of Ecofriendly Derivative Spectrophotometric Methods for the Simultaneous Quantitative Analysis of Remogliflozin and Vildagliptin from Formulation
Source: Molecules. 2021 Oct 12;26(20):6160. doi: 10.3390/molecules26206160 (PMC8537597; doi:10.3390/molecules26206160)
Supplement: Supplementary file 1 [file molecules-26-06160-s001.zip › molecules-1397718-supplementary/Suplementary file/File S1.pdf]

Calibration Curves for Remogliflozin and Vildagliptin

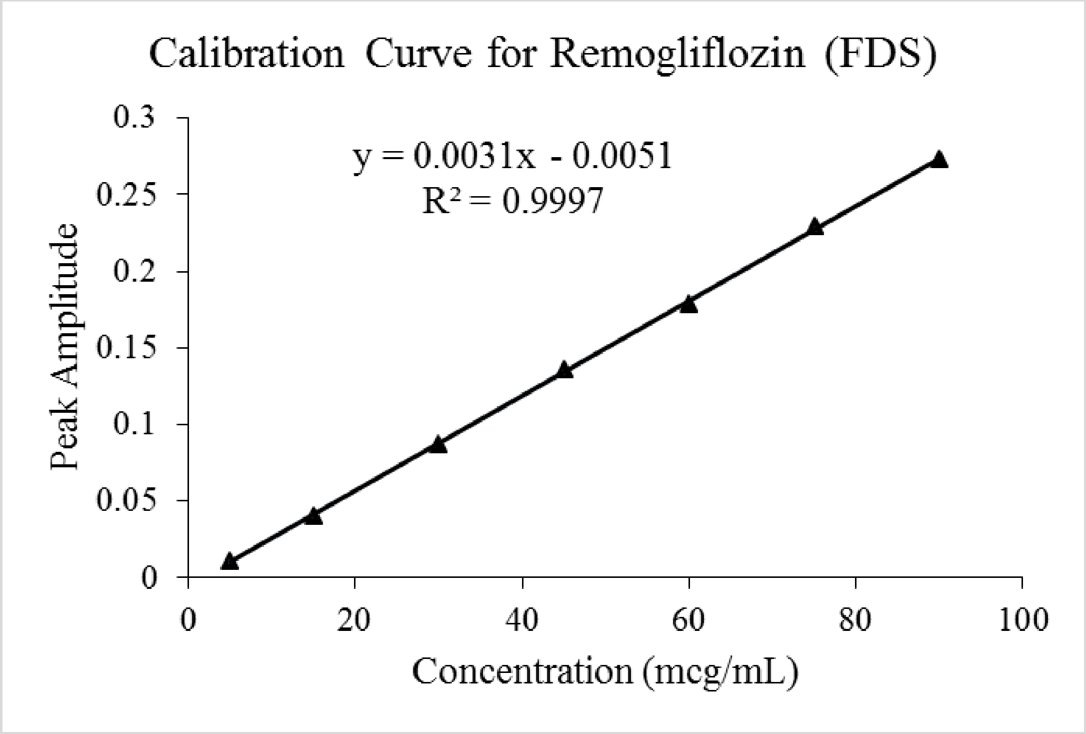


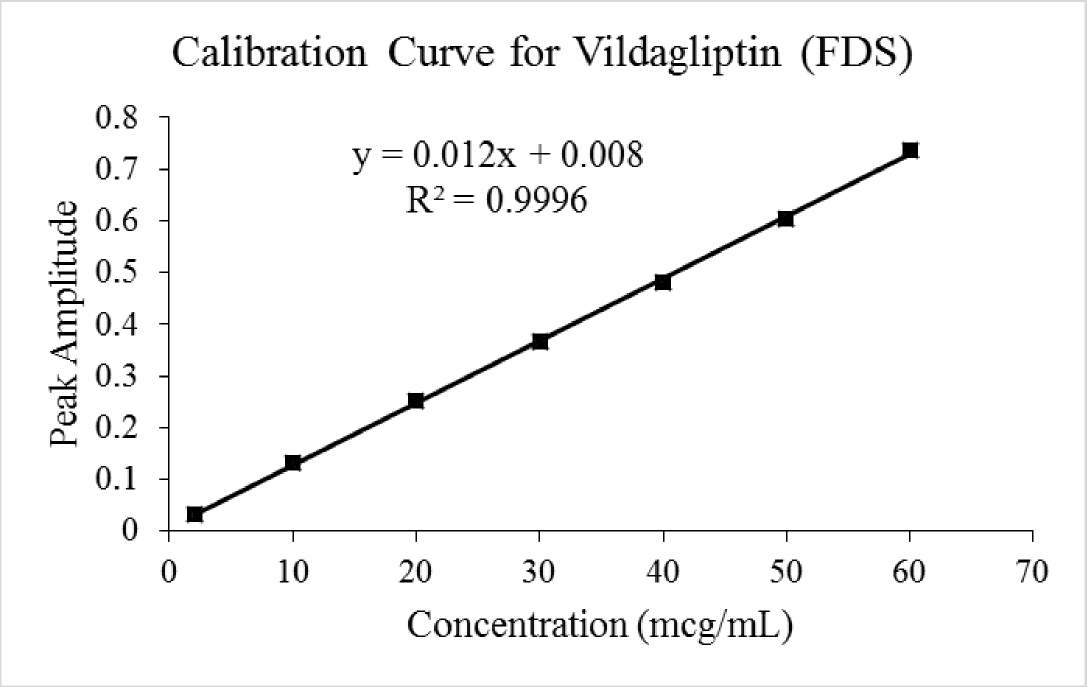

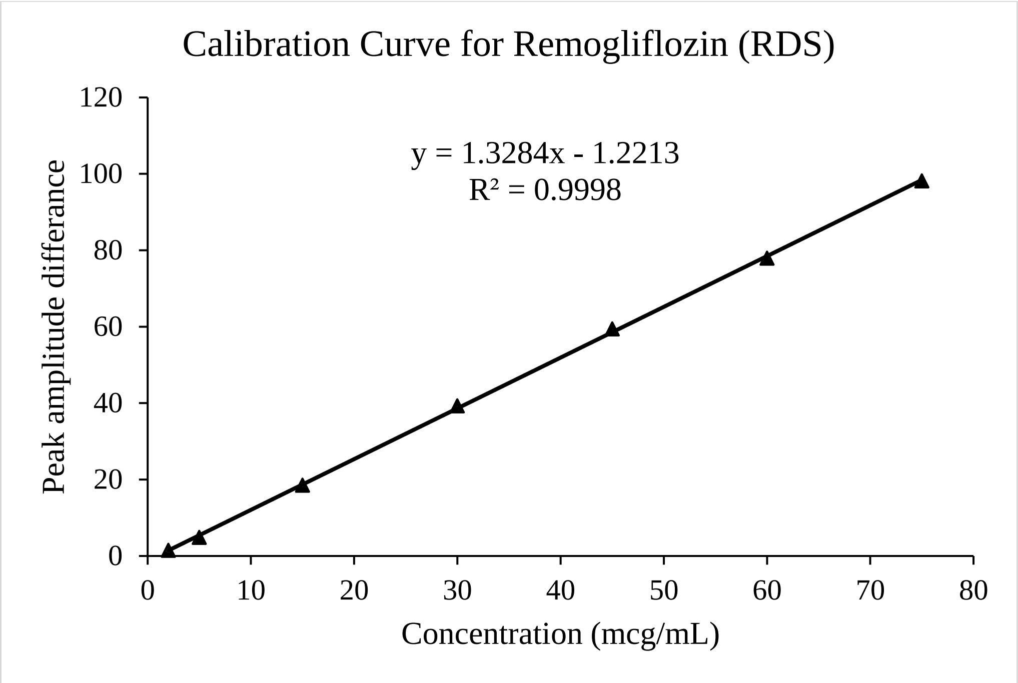
Calibration Curve for Remogliflozin (RDS)
y = 1.3284x - 1.2213
R² = 0.9998
Peak amplitude differance
Concentration (mcg/mL)
0
20
40
60
80
100
120
0
10
20
30
40
50
60
70
80

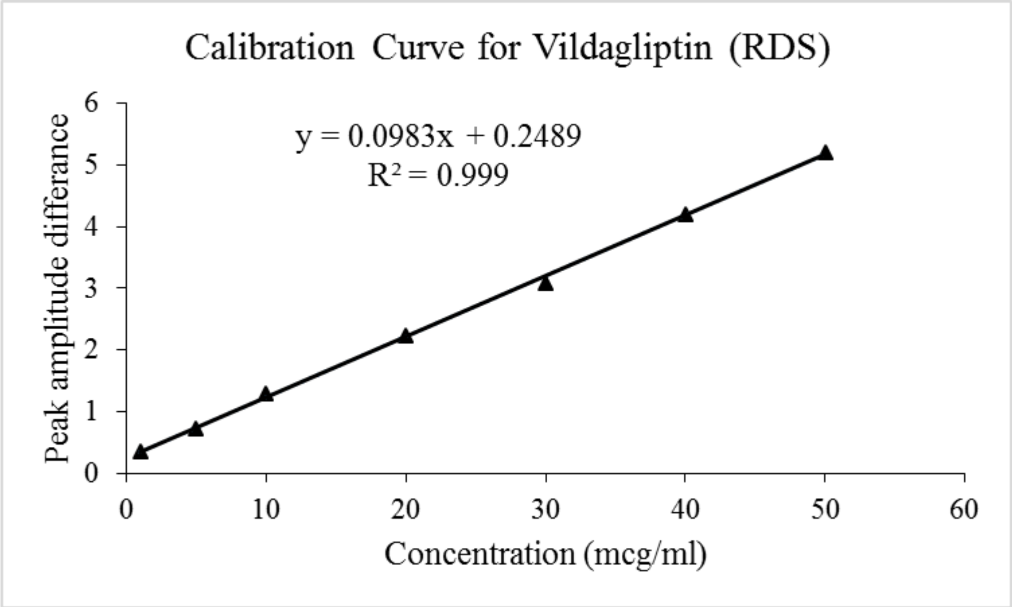
Calibration Curve for Vildagliptin (RDS)
y = 0.0983x + 0.2489
R² = 0.999
Peak amplitude differance
Concentration (mcg/ml)
0
1
2
3
4
5
6
0
10
20
30
40
50
60

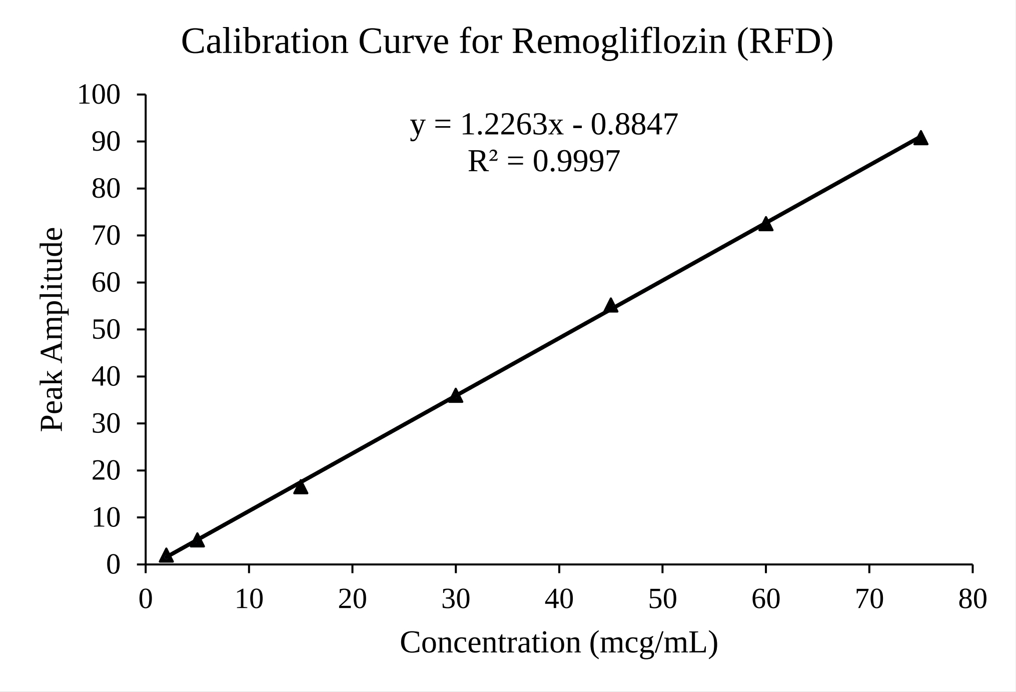

Calibration Curve for Remogliflozin (RFD)
y = 1.2263x - 0.8847
R² = 0.9997
Peak Amplitude
Concentration (mcg/mL)
0
10
20
30
40
50
60
70
80
90
100


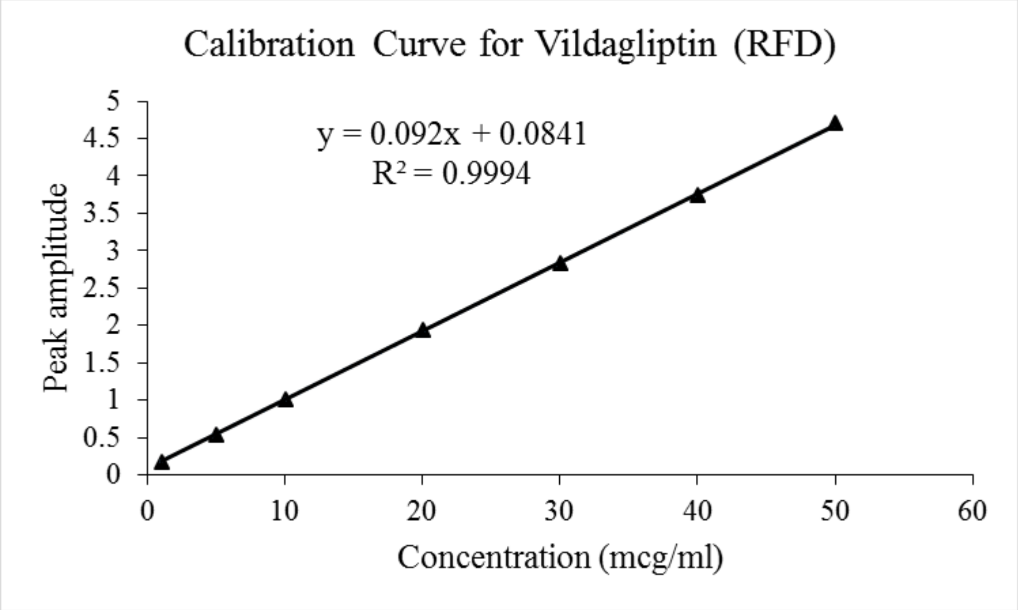

Calibration Curve for Vildagliptin (RFD)
y = 0.092x + 0.0841
R² = 0.9994
Peak amplitude
Concentration (mcg/ml)
0
0.5
1
1.5
2
2.5
3
3.5
4
4.5
5
10
20
30
40
50
60
