# Supplementary material for: Development of Ecofriendly Derivative Spectrophotometric Methods for the Simultaneous Quantitative Analysis of Remogliflozin and Vildagliptin from Formulation
Source: Molecules. 2021 Oct 12;26(20):6160. doi: 10.3390/molecules26206160 (PMC8537597; doi:10.3390/molecules26206160)
Supplement: Supplementary file 1 [file molecules-26-06160-s001.zip › molecules-1397718-supplementary/Suplementary file/Supplementary Table S1 and S2.pdf]

Supplementary Material

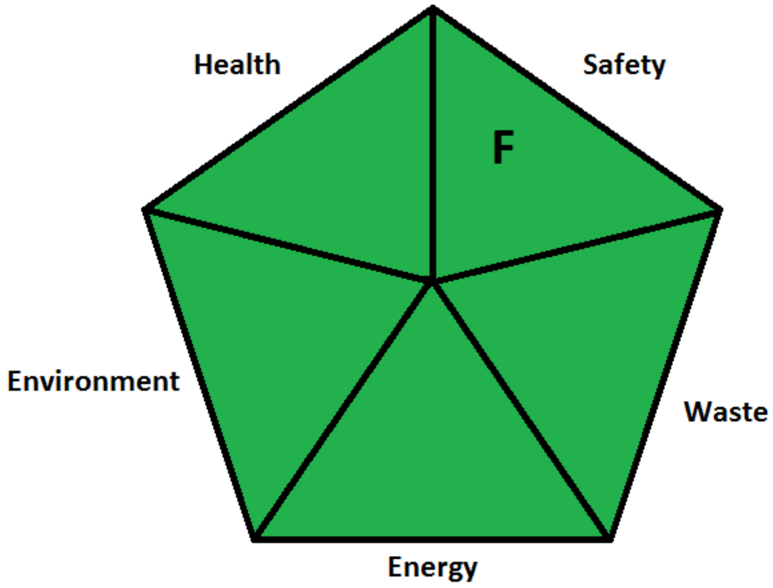

Category	Green	Yellow	Red
**Health Hazard**	Slightly toxic, slight irritant; NFPA health hazard score is 0 or 1.	Moderately toxic; could cause temporary incapacitation; NFPA = 2 or 3.	Serious injury on short term exposure; known or suspected small animal carcinogen; NFPA = 4.
**Safety Hazard**	Highest NFPA flammability, instability score of 0 or 1. No special hazards.	Highest NFPA flammability or instability score is 2 or 3, or a special hazard is used.	Highest NFPA flammability or instability score is 4.
**Environmental Hazard**	If less than 50 g of environmental hazards used.	If more than 50 g but less than 250 g used.	If more than 250 g used.
**Energy**	Wet chemistry method such as titration. Very little solvent evaporation.	Instrumental method such as GC, HPLC; moderate solvent evaporation.	Instrumental method such as GC-MS; high volume of solvent evaporated.
**Waste amount**	Total waste for processing one sample ≤ 50 g.	Total waste ≤ 250 g.	Total waste > 250 g.

Table S1. Green assessment profile proposed by Raynie et al. [1]

References

D. Raynie, J. Driver, Green Assessment of Chemical Methods, In: 13th Annual Green Chemistry and Engineering Conference, *Maryland*, 2009.

Table S2: Green analytical procedure Index parameters [2]

Category	Green	Yellow	Red
**Sample preparation**			
Collection (1)	In-line	On-line or at-line	Off-line
Preservation (2)	None	Chemical or physical	Physico-chemical
Transport (3)	None	Required	–
Storage (4)	None	Under normal conditions	Under special conditions
Type of method: direct or indirect (5)	No sample preparation	Simple procedures, eg. filtration, decantation	Extraction required
Scale of extraction (6)	Nano-extraction	Micro-extraction	Macro-extraction
Solvents/reagents used (7)	Solvent-free methods	Green solvents/reagents used	Non-green solvents/reagents used
Additional treatments (8)	None	Simple treatments (clean up, solvent removal, etc.)	Advanced treatments (derivatization, mineralization, etc.)
**Reagent and solvents**			
Amount (9)	< 10 mL (< 10 g)	10–100 mL (10–100 g)	> 100 mL (> 100 g)
Health hazard (10)	Slightly toxic, slight irritant; NFPA health hazard score = 0 or 1.	Moderately toxic; could cause temporary incapacitation; NFPA = 2 or 3.	Serious injury on short-term exposure; known or suspected small animal carcinogen; NFPA = 4.
Safety hazard (11)	Highest NFPA flammability or instability score of 0 or 1. No special hazards.	Highest NFPA flammability or instability score of2 or 3, or a special hazard is used.	Highest NFPA flammability or instability score of 4.
**Instrumentation**			
Energy (12)	≤0.1 kWh per sample	≤1.5 kWh per sample	> 1.5 kWh per sample
Occupational hazard (13)	Hermetic sealing of analytical process	–	Emission of vapours to the atmosphere
Waste (14)	< 1 mL (< 1 g)	1–10 mL (1–10 g)	> 10 mL (< 10 g)
Waste treatment (15)	Recycling	Degradation, passivation	No treatment
**ADDITIONAL MARK: QUANTIFICATION**			
Circle in the middle of GAPI: *Procedure for qualification and quantification*		No circle in the middle of GAPI: *Procedure only for qualification*	
NFPA: National Fire Protection Association			

References

1 Van Aken, K.; Strekowski, L.; Patiny, L. EcoScale, A semi-quantitative tool to select an organic preparation based on economical and ecological parameters. *Beilstein J. Org. Chem.* **2006**, *2*, 1–7.

2 Płotka-Wasylka, J. A new tool for the evaluation of the analytical procedure: Green Analytical Procedure Index. *Talanta* **2018**, *181*, 204–209.
