# Supplementary figures and images for: Development of Ecofriendly Derivative Spectrophotometric Methods for the Simultaneous Quantitative Analysis of Remogliflozin and Vildagliptin from Formulation
Source: Molecules. 2021 Oct 12;26(20):6160. doi: 10.3390/molecules26206160 (PMC8537597; doi:10.3390/molecules26206160)

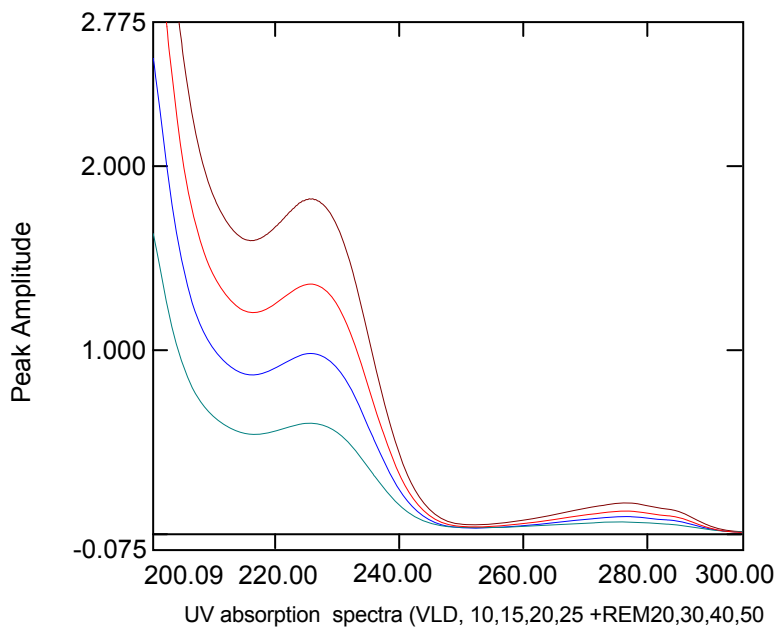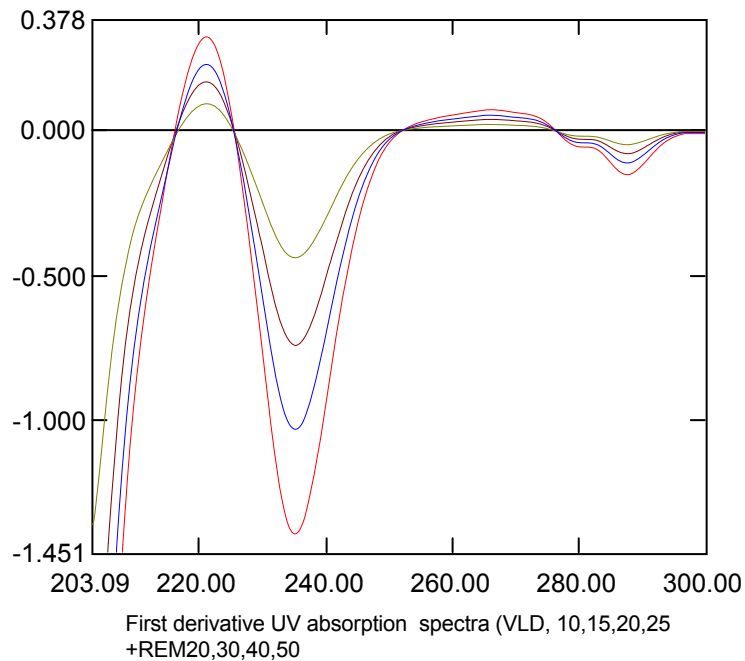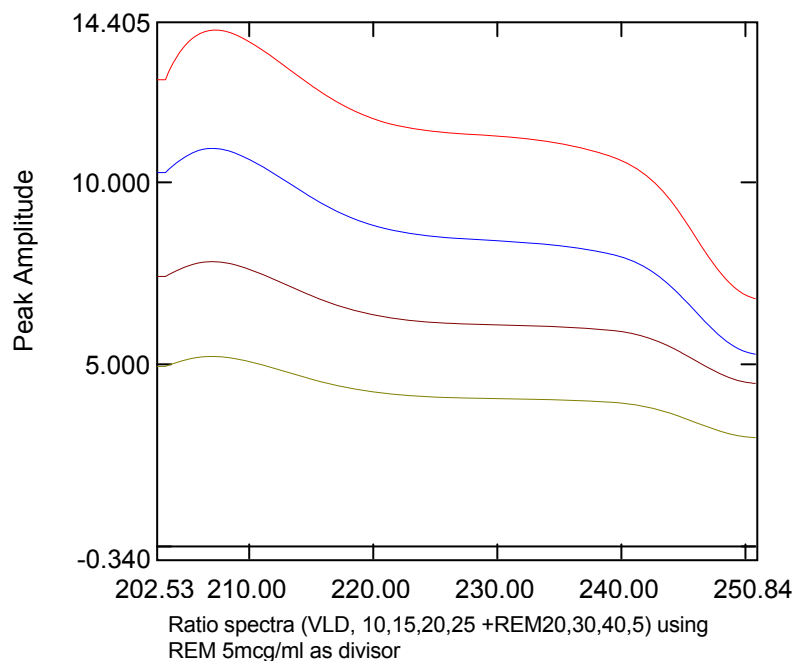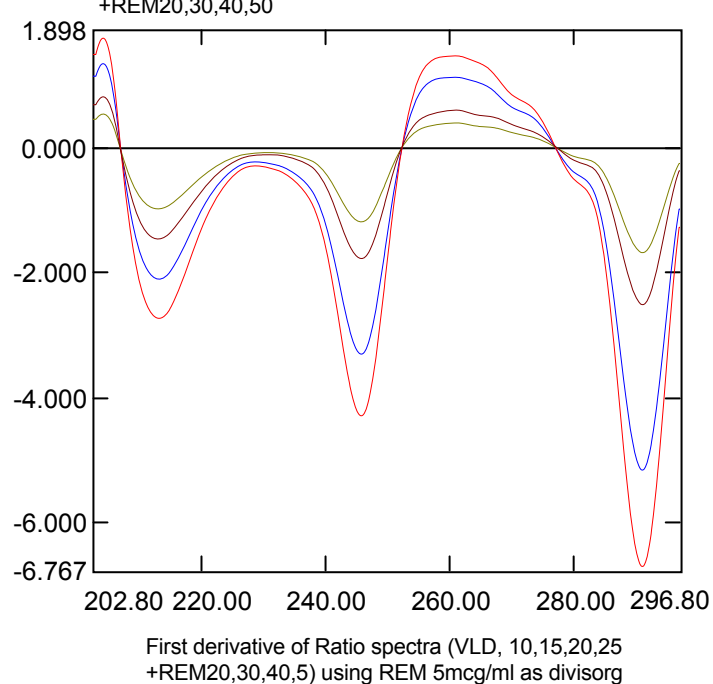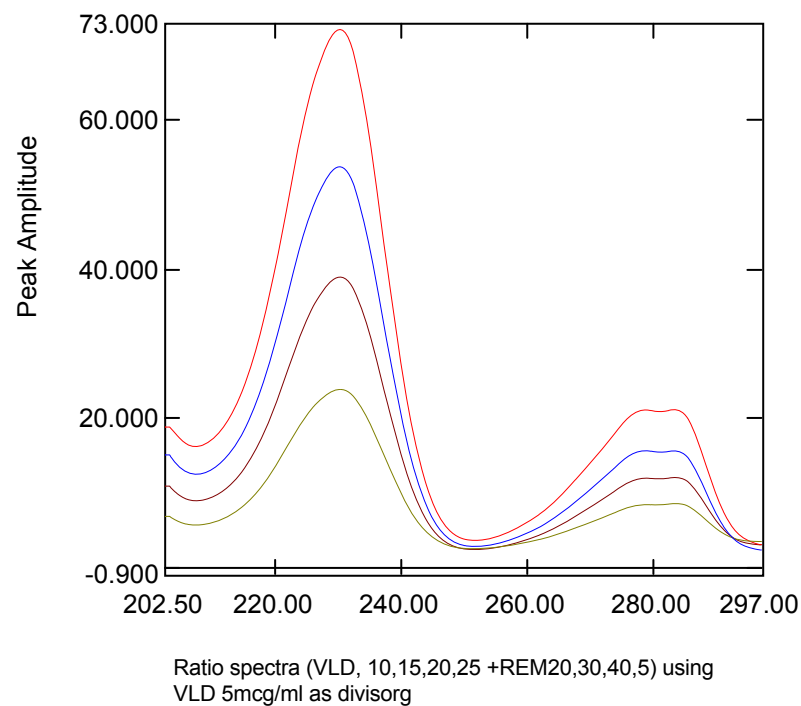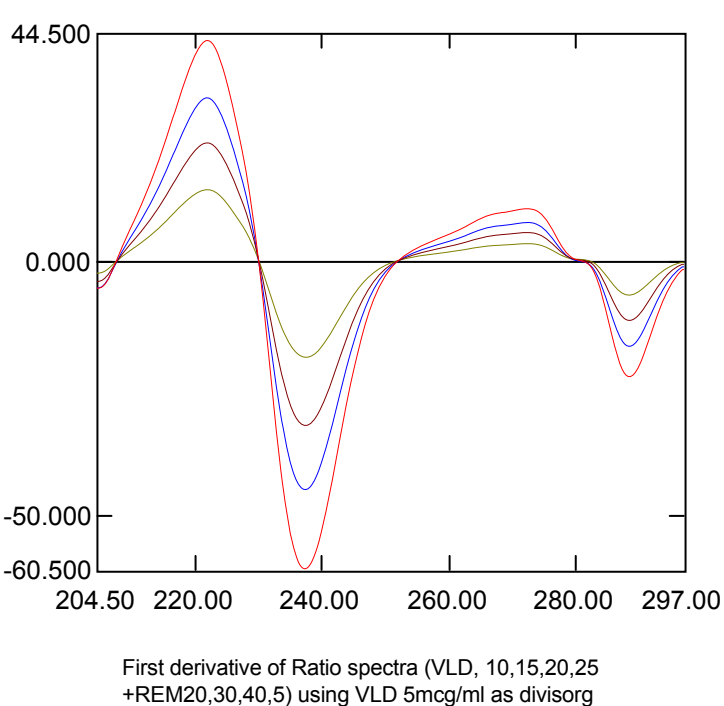

Supplement: Supplementary file 1 [file molecules-26-06160-s001.zip › molecules-1397718-supplementary/Suplementary file/Figure S2.pdf]
